# Association of antibodies to *Plasmodium falciparum* reticulocyte binding protein homolog 5 with protection from clinical malaria

**DOI:** 10.3389/fmicb.2014.00314

**Published:** 2014-06-30

**Authors:** Chris Y. H. Chiu, Julie Healer, Jennifer K. Thompson, Lin Chen, Aiki Kaul, Laxman Savergave, Arjun Raghuwanshi, Connie S. N. Li Wai Suen, Peter M. Siba, Louis Schofield, Ivo Mueller, Alan F. Cowman, Diana S. Hansen

**Affiliations:** ^1^The Walter and Eliza Hall Institute of Medical ResearchParkville, VIC, Australia; ^2^Department of Medical Biology, The University of MelbourneParkville, VIC, Australia; ^3^Gennova BiopharmaceuticalsPune, India; ^4^Vector Borne Disease Unit, Papua New Guinea Institute of Medical ResearchGoroka, New Guinea; ^5^Australian Institute of Tropical Health and Medicine, James Cook UniversityDouglas, QLD, Australia; ^6^Barcelona Center for International Health, University of BarcelonaBarcelona, Spain

**Keywords:** malaria, *Plasmodium falciparum*, reticulocyte binding protein homolog 5, antibodies, immunity

## Abstract

Emerging evidence suggests that antibodies against merozoite proteins involved in *Plasmodium falciparum* invasion into the red blood cell (RBC) play an important role in clinical immunity to malaria. The protein family of parasite antigens known as *P. falciparum* reticulocyte binding protein-like homolog (PfRh) is required for RBC invasion. PfRh5 is the only member within the PfRh family that cannot be genetically deleted, suggesting it plays an essential role in parasite survival. This antigen forms a complex with the cysteine-rich *P. falciparum* Rh5 interacting protein (PfRipr), on the merozoite surface during RBC invasion. The PfRh5 ectodomain sequence and a C-terminal fragment of PfRipr were cloned and expressed in *Escherichia coli* and baculovirus-infected cells, respectively. Immunization of rabbits with these recombinant proteins induced antibodies able to inhibit growth of various *P. falciparum* strains. Antibody responses to these proteins were investigated in a treatment–re-infection study conducted in an endemic area of Papua New Guinea (PNG) to determine their contribution to naturally acquired immunity. Antibody titers to PfRh5 but not PfRipr showed strong association with protection against *P. falciparum* clinical episodes. When associations with time-to-first infection were analyzed, high antibody levels against PfRh5 were also found to be associated with protection from high-density infections but not from re-infection. Together these results indicate that PfRh5 is an important target of protective immunity and constitutes a promising vaccine candidate.

## INTRODUCTION

Malaria is one of the most serious infectious diseases of humans causing 500 million clinical cases annually, with nearly 25% of the global burden occurring in the Asia-Pacific. The blood stage of the *Plasmodium* parasite is entirely responsible for malaria-associated pathology (Miller et al., 2002). Fatalities are associated with a spectrum of disease syndromes including acute respiratory distress, hypoglycemia, renal failure, pulmonary oedema and cerebral involvement. The most susceptible population to severe malaria are children under the age of 5, who have experienced few parasitic infections. After years of repeated exposure, individuals living in endemic areas develop clinical immunity. This form of protection does not result in sterilizing immunity but prevents clinical episodes by significantly reducing parasite burden. Naturally acquired immunity predominantly targets blood-stage parasites and appears to require antibody responses since passive transfer of sera from clinically immune individuals protects non-immune recipients from high parasitemia and disease symptoms (Cohen et al., 1961).

During blood-stage replication, *Plasmodium falciparum* merozoites invade erythrocytes through a complex multistep process that requires initial contact of the parasite with the red blood cell (RBC) surface followed by apical reorientation of the merozoite, tight junction formation and final entry into the erythrocyte (reviewed in Cowman and Crabb, 2006). These invasion steps depend on interactions between specific parasite proteins and their receptors on the erythrocyte surface. Two families of invasion ligands have been identified in *P. falciparum*: the erythrocyte-binding antigens (EBAs) and the *P. falciparurm* reticulocyte binding protein-like homologs (PfRhs; reviewed in Cowman and Crabb, 2006). EBAs are orthologs of the Duffy-binding protein of *P. vivax* and include EBA-140, EBA-175, and EBA-181. They consist of an N-terminal cysteine-rich domain, a highly conserved domain, a C-terminal cysteine-rich domain and a transmembrane and cytoplasmic domain (reviewed in Cowman and Crabb, 2006). EBAs are located in the micronemes and are secreted onto the parasite surface just before invasion. Whereas EBA-175 has been shown to interact with glycophorin A on the surface of the erythrocyte (ref), EBA-140 binds to glycophorin C (Maier et al., 2009). The receptor for EBA-181 has not been identified. The PfRhs family consists of five proteins located in the parasite’s rhoptries. Members of this family include: PfRh1, PfRh2a, PfRh2b, PfRh4, and PfRh5. So far only the host receptor for PfRh4 (Tham et al., 2010; complement receptor 1) and PfRh5 (Crosnier et al., 2011; basigin) have been identified. Except for PfRh5 (Baum et al., 2009), all the other members of this family are large type-1 transmembrane proteins.

PfRh5 is considerably smaller than the other PfRh proteins and lacks a transmembrane domain (Baum et al., 2009). After its release from the rhoptries, PfRh5 forms a complex with a cysteine-rich antigen named *P. falciparum* Rh5 interacting protein (PfRipr), which facilitates its expression on the merozoite’s surface for erythrocyte invasion (Chen et al., 2011). The genes encoding both PfRh5 and PfRipr are refractory to gene targeted deletion, suggesting essential roles for these antigens in parasite invasion (Baum et al., 2009; Chen et al., 2011).

*P. falciparum* invasion ligands are targets of inhibitory antibodies that prevent parasite invasion and subsequent replication in the erythrocyte (reviewed in Cowman and Crabb, 2006). Thus these molecules have been proposed as vaccine candidates. With this in view, PfRh5 has recently received considerable attention, since unlike many other merozoite antigens, it has limited genetic diversity among *P. falciparum* isolates (Bustamante et al., 2013). Moreover, rabbit antisera raised against PfRh5 have been shown to inhibit parasite replication *in vitro* (Baum et al., 2009; Douglas et al., 2011). Parasite growth inhibition was observed across a wide range of laboratory-adapted parasite lines, suggesting that PfRh5 could be an effective vaccine target (Bustamante et al., 2013; Douglas et al., 2014; Reddy et al., 2014). Similarly, antibodies to PfRipr have been shown to inhibit parasite growth *in vitro* (Chen et al., 2011).

To date, it is unclear whether PfRipr is recognized by naturally acquired antibodies from individuals residing in malaria-endemic areas. Moreover, despite promising results in experimental animals and *in vitro* assays on the potential for PfRh5 as a leading vaccine candidate, there are conflicting reports on whether this antigen is the target of naturally acquired immunity, with studies suggesting suggesting that PfRh5 has poor natural immunogenicity (Douglas et al., 2011) or that anti-PfRh5 responses predict protection from clinical malaria (Tran et al., 2014). To further address this question, we have expressed stable, soluble forms of PfRh5 and PfRipr. The association between antibody responses to the PfRh5 and PfRipr recombinant proteins with reduced risk to re-infection and symptomatic disease was investigated in a treatment–reinfection study in a malaria-endemic area. Our main results indicate that in a population of children who are actively acquiring immunity to malaria, anti-PfRh5 antibody responses are associated with protection against *P. falciparum* clinical episodes and high-density infections.

## MATERIALS AND METHODS

### STUDY POPULATION AND ETHICS STATEMENT

Plasma samples were obtained from a prospective treatment–reinfection study of 206 children aged from 5 to 14 years conducted in Madang province of Papua New Guinea (PNG). Full details of this cohort have been previously described (Michon et al., 2007). Briefly, venous blood was collected at study enrolment into heparinized tubes and plasma was stored at -80^∘^C. All participants received a 7-day treatment of oral artesunate to clear existing infections. During a 6-month follow up period, participants were monitored every two weeks for symptomatic illness and/or parasitemia (active surveillance) and when a child presented with symptoms at the local Mugil Health Centre (passive surveillance). Re-infection was detected by post-PCR ligase detection reaction–fluorescent microsphere assay (LDR-FMA) and light microscopy (LM) on Giemsa stained blood smears. Re-infections were categorized to (1) PCR-detectable, (2) LM-detectable, (3) LM-detectable re-infection with >500 parasites per μl, and (4) LM-detectable re-infection with >5000 parasites per μl. Clinical episode was defined as the presence of fever with ≥37.5^∘^C and >5000 parasites/μl. The study was approved by the Medical Research Advisory Committee (MRAC), PNG Ministry of Health, The Walter and Eliza Hall Institute Human Research Ethics Committee and the institutional review board of the Veteran’s Affairs Medical Center (Cleveland, OH, USA). Written consent was obtained from parents/guardians of all participants.

### EXPRESSION OF RECOMBINANT PROTEINS AND ANTIBODY GENERATION

The PfRh5 ectodomain sequence was codon-optimized for *Escherichia coli* expression and cloned into the pET-303 vector (Invitrogen, MA, USA). The C-terminally HIS tagged expression product was refolded from the insoluble pellet material under standard conditions. PfRipr (AA604-1086) was cloned into pTri-Ex 2 vector for expression in baculovirus-infected Hi-5 cells (Life Technologies, MA, USA). HIS-FLAG tagged PfRiPr protein was purified from the cell culture supernatant by incubation with M2-FLAG beads (Sigma, MO, USA), followed by elution with FLAG peptide in NaCl-Tris buffer pH 8.0. The peptide was removed by dialysis prior to immunization. Purity and integrity of recombinant proteins were assessed in SDS–polyacrylamide gels (Invitrogen, MA, USA). Rabbits were immunized twice with 100 μg PfRh5 in GLA-SE adjuvant (a kind gift from Darrick Carter, IDRI), or three times with 200 μg PfRipr in Freund’s adjuvant. Total IgG was purified from immune sera as described (Healer et al., 2013). All experiments were performed in compliance with the Walter and Eliza Hall Institute Animal Ethics Committee requirements.

### GROWTH INHIBITION ASSAYS

Growth inhibition assays (GIA) using IgG isolated from immunized rabbits were performed as described (Healer et al., 2013). Briefly, serial dilutions of purified IgG starting at 2 mg/ml were added to *P. falciparum-*infected RBC (3D7, W2mef, and FCR3) at a parasitemia of 0.5%. Parasitemia was counted after 48 h and specific growth inhibition calculated relative to parasites grown in non-immune IgG. Specific growth inhibition was calculated relative to parasites grown in non-immune IgG.

### ELISA

Microtiter plates were coated with recombinant PfRh5 (2 μg/ml) and PfRipr (0.5 μg/ml) in carbonate buffer pH 9.6 by overnight incubation at 4^∘^C. Empty sites were blocked with 5% skim milk for 1 h at 37^∘^C. After washing with 0.05% Tween-PBS, 100 μl of 1:2 serially diluted plasma samples were added to plates and incubated at 37^∘^C for 1 h. The plates were washed three times and incubated with a peroxidase-conjugated mouse anti-human antibody (Southern Biotech, AL, USA). Bound complexes were detected by reaction with tetramethy-benzidine (KBL, MD, USA) and H_2_O_2_. Absorbance was read at 450 nm. Plasma samples from malaria naïve anonymous Australian blood donors were included as negative controls. Antibody titers were calculated as the plasma dilution with an optical density (OD) value higher than that observed for negative controls at a 1/100 dilution.

### STATISTICAL ANALYSES

Statistical analysis was performed using STATA 9.2 (STATA-Corp., College Station, TX, USA). For analysis of associations with clinical outcomes, participants with no detectable parasitemia by PCR or LM during follow up were regarded as non-exposed and excluded from the analysis. Demographic and clinical variables were assessed as potential confounders. Kaplan–Meier method and log-rank test were used to explore associations between antibody titers and time to *P. falciparum* re-infection or clinical episodes. Cox proportional hazards modeling was used to calculate hazard ratios (HR) for time-to-first *P. falciparum* infection by PCR, LM, and time-to-first infection of >500 and >5000 parasites/μL. Poisson model was used to obtain incidence rate ratio (IRR) for the incidence of clinical malaria episodes throughout the study period. HR and IRR were adjusted for identified confounders. Differences in antibody titers between categorical variables were assessed using Wilcoxon rank sum test or Kruskal–Wallis tests.

## RESULTS

### RECOMBINANT PfRh5 AND PfRipr ARE IMMUNOGENIC AND INDUCE PARASITE GROWTH INHIBITORY ANTIBODIES

**FIGURE 1:**
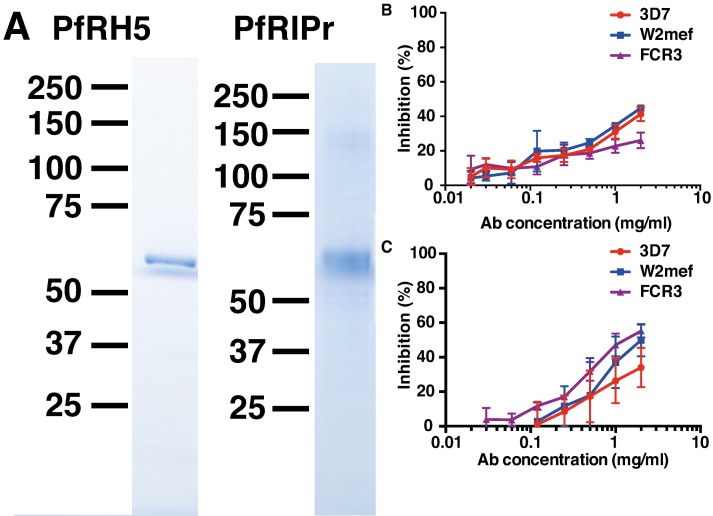
**Immunization of rabbits with recombinant PfRh5 and PfRip5 induces strain-transcending parasite growth inhibitory antibodies.** The PfRh5 ectodomain sequence and the C-terminal PfRipr fragment were cloned and expressed in *E. coli* and baculovirus-infected Hi-5 cells, respectively. SDS-PAGE analysis **(A)** of the purified recombinant proteins. Total IgG purified from sera of PfRh5 **(B)** and PfRipr **(C)** immunized rabbits was added to *P. falciparum* 3D7, W2Mef, and FCR3 cultures to determine their capacity to inhibit parasite growth.

The PfRh5 ecotodomain sequence and the C-terminal PfRipr fragment from 3D7 were cloned and expressed in *E. coli* and baculovirus-infected insect cells, respectively. HIS-FLAG tagged proteins were purified with M2-FLAG beads. SDS-PAGE analysis of the purified recombinant proteins revealed highly pure protein preparations with predominant bands at 63 kDa corresponding to PfRh5 and 65 kDa corresponding to PfRipr (**Figure 1A**).

To determine if the recombinant proteins were immunogenic, rabbits were immunized twice with 100 μg PfRh5 in GLA-SE adjuvant or three times with 200 μg PfRiPr in Freund’s adjuvant. Seroconversion of immunized rabbits was successfully confirmed by ELISA using PfRh5 and PfRipr recombinant proteins (data not shown). Total IgG was purified from immune sera 2 weeks after the last immunization and added to *P. falciparum* trophozoite cultures to determine their capacity to inhibit parasite growth. **Figures 1B,C** shows that both anti-PfRh5 and anti-PfRipr antibodies inhibit growth of *P. falciparum* 3D7, W2Mef, and FCR3 in a dose dependent manner. Incubation with 2 mg/ml of both antibodies resulted in ∼40% of growth inhibition of all parasite strains tested. Thus immunization with these novel recombinant proteins induces antibodies that prevent growth of multiple *P. falciparum* clones.

### ANTI-PfRh5 ANTIBODIES ARE ASSOCIATED WITH PROTECTION FROM HIGH PARASITEMIA AND REDUCE INCIDENCE OF MALARIA CLINICAL EPISODES

**FIGURE 2:**
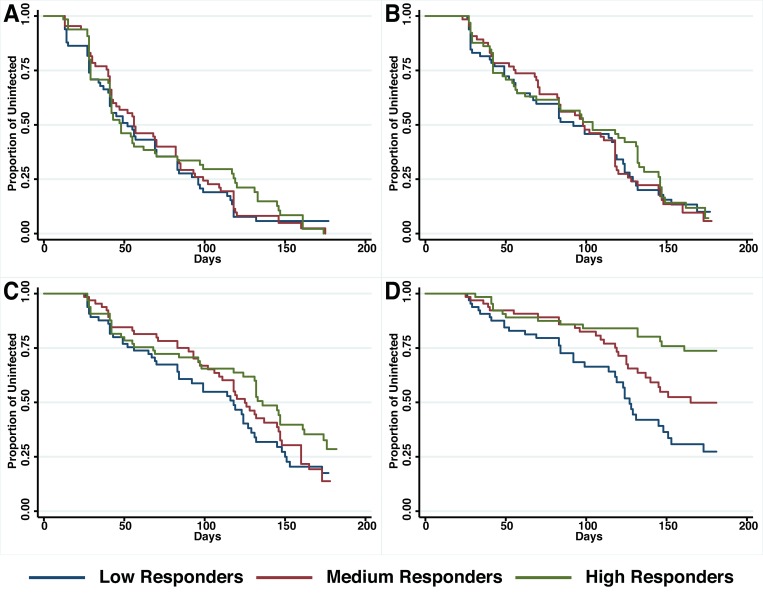
**Probability of remaining free of malaria re-infection amongst individuals with different anti-PfRh5 antibody levels.** Time-to-first PCR-positive *P. falciparum* blood-stage infection **(A)**, LM-positive **(B)**, LM-positive with ≥500 parasites/μL **(C)** and LM-positive with ≥5000 parasites/μl **(D)** in individuals with low, medium or high anti-PfRH5-specific antibody titers at study enrolment, as determined by Kaplan–Meier survival analysis. Log-rank was used to determine differences among responder groups, *p* < 0.0001 between high and low responders and *p* = 0.019 between medium and low responders with LM ≥ 5000 parasites/μl.

A preliminary screen conducted in the Madang Province of PNG suggested that antibodies to PfRh5 and to the EGF-like domain of PfRipr show an intermediate association with protection from clinical malaria (Richards et al., 2013). To further explore this hypothesis, antibody titers to the recombinant PfRh5 and PfRipr proteins described above, were measured by ELISA in a longitudinal cohort of PNG children. Similar to other endpoints previously examined in this cohort (Michon et al., 2007; Richards et al., 2010; Hill et al., 2013), antibody responses to PfRh5 were heterogeneous and thus divided into terciles for analysis. The relationship between antibody levels amongst low (L), medium (M), and high (H) responders and time-to-reinfection of different parasite densities was investigated in Kaplan–Meier survival curves and a log-rank test was used to determine the difference between groups. **Figure 2** shows that anti-PfRh5 responses protected from high-density (≥5000 parasites/μL) parasitemia (H vs L: *p* < 0.0001, M vs L: *p* = 0.019) but not against re-infection *per se* (assessed by PCR or LM). The association between antibody titers and the risk of acquiring new *P. falciparum* infections was also analyzed by Cox regression model. In agreement with previous studies with this cohort (Michon et al., 2007; Richards et al., 2010; Hill et al., 2013), age (<9 years and ≥9 years) and location of residence were identified as confounders. Thus HRs were adjusted for those variables. Anti-PfRh5 antibody responses did not reduce the risk of *P. falciparum* re-infection by PCR, LM or moderate density infection (>500 parasites/μL). However, a reduced risk of developing a high-density infection (>5000 parasites/μL) was observed for high anti-PfRh5 titers, even after adjustment for age and location of participants (**Table 1**).

**Table 1:** Associations between anti-PfRh5 antibody titers and risk of acquiring *P. falciparum* infections of varying density.

		Anti-PfRh5 responses					
		Medium vs low			High vs low		
		HR	95% CI	*P*	HR	95% CI	*P*
PCR	Unadjusted	0.95	(0.66–1.35)	0.76	0.88	(0.61–1.27)	0.51
PCR	Adjusted	0.98	(0.68–1.42)	0.93	0.87	(0.57–1.33)	0.53
LM	Unadjusted	0.99	(0.68–1.45)	0.96	0.89	(0.61–1.31)	0.56
LM	Adjusted	1.10	(0.74–1.64)	0.64	1.05	(0.67–1.65)	0.83
LM500	Unadjusted	0.87	(0.58–1.30)	0.49	**0.63**	**(0.41–0.97)**	**0.03**
LM500	Adjusted	1.00	(0.64–1.54)	0.99	0.85	(0.51–1.42)	0.55
LM5000	Unadjusted	**0.56**	**(0.34–0.93)**	**0.03**	**0.27**	**(0.15–0.50)**	**<0.001**
LM5000	Adjusted	0.61	(0.36–1.05)	0.07	**0.37**	**(0.18–0.72)**	**<0.001**

*Hazard ratio (HRs) were determined to assess associations with anti-PhRh5 antibody responses and time-to-first PCR detectable, LM detectable, ≥500 parasites/μl, and ≥5000 parasites/μl *P. falciparum* infections. HRs were adjusted for age and location of participants. Significant associations are shown in bold.*

**FIGURE 3:**
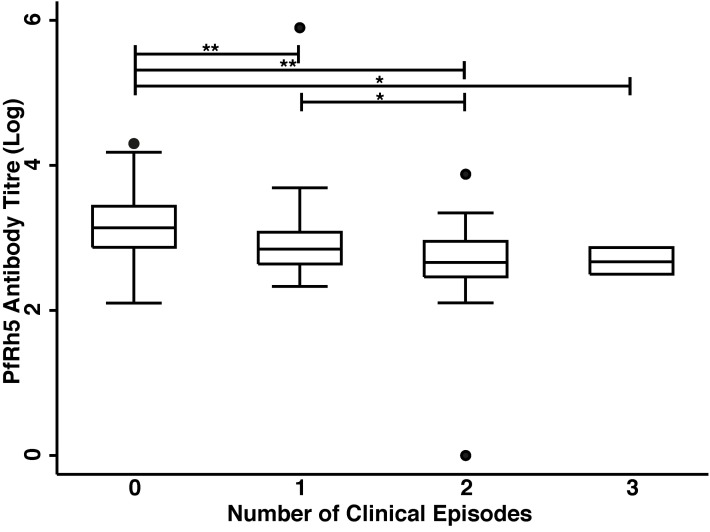
**High anti-PfRh5 antibody levels in individuals protected from clinical malaria.** Anti-PfRh5 antibody titers were stratified on the basis of the number of clinical episodes during the 6 month follow-up period. Statistical significance was determined by Kruskal–Wallis test and by Wilcoxon rank sum test (*p < 0.01, **p < 0.001).

The association of anti-PfRh5 antibody responses with time-to-first clinical episode was also determined. Anti-PfRh5 antibody levels were found to be significantly associated with a protection from clinical malaria, as medium and high antibody responders showed 61.7 and 82.2% reduced risk of experiencing a clinical episode than low responders after the adjustment for confounders (M vs L: adjusted HR = 0.383, 95% CI = 0.216–0.678, *p* = 0.001; H vs L: adjusted HR = 0.178, 95% CI = 0.082–0.389, *p* < 0.001; **Table 2**). A Poisson regression was used to test for associations between antibody levels and overall incidence of clinical disease. Increased anti-PfRh5 antibody responses were found to be strongly associated with reduced incidence of malaria clinical episode during the 6 month follow-up period (M vs L: adjusted IRR = 0.539, 95% CI = 0.344–0.842, *p* = 0.007; H vs L: adjusted IRR = 0.257, 95% CI = 0.134–0.492, *p* < 0.001; **Table 3**). Moreover, anti-PfRh5 antibody titers were found to be significantly higher in individuals that did not experience clinical episodes compared to those that experienced 1, 2, or 3 malaria clinical episodes over the follow up period (**Figure 3**). Thus together these results indicate that naturally acquired anit-PfRh5 antibodies protect from parasitemia of high density and clinical illness.

**Table 2:** Association between IgG responses to PfRh5 and PfRipr and time-to-first clinical episode.

	PfRh5						PfRipr					
	M vs L			H vs L			M vs L			H vs L		
	HR	95% CI	*P*	HR	95% CI	*P*	HR	95% CI	*P*	HR	95% CI	*P*
Unadjusted	**0.45**	**(0.26–0.77)**	**<0.01**	**0.17**	**(0.08–0.35)**	**<0.01**	0.90	(0.51–1.60)	0.73	0.55	(0.30–1.00)	0.053
Adjusted	**0.38**	**(0.22–0.68)**	**<0.01**	**0.18**	**(0.08–0.39)**	**<0.01**	0.90	(0.50–1.61)	0.72	0.64	(0.35–1.17)	0.150

*Hazard ratios (HRs) were determined to assess associations with anti-PhRh5 and anti-PfRipr antibody responses and time to *P. falciparum* clinical episodes with ≥5000 parasites/μl. Values represent unadjusted and adjusted ratios (age and location) ±95% confidence intervals (CI). Significant associations are shown in bold.*

**Table 3:** Association between IgG responses to PfRh5 and PfRipr and incidence of malaria clinical episodes.

	PfRh5						PfRipr					
	M vs L			H vs L			M vs L			H vs L		
	IRR	95% CI	*P*	IRR	95% CI	*P*	IRR	95% CI	*P*	IRR	95% CI	*P*
Unadjusted	**0.55**	**(0.35–0.85)**	**<0.01**	**0.21**	**(0.12–0.39)**	** <0.01**	1.03	(0.64–1.65)	0.90	0.81	(0.49–1.33)	0.41
Adjusted	**0.54**	**(0.34–0.84)**	**<0.01**	**0.26**	**(0.13–0.49)**	** <0.01**	1.05	(0.65–1.69)	0.83	0.87	(0.53–1.44)	0.58

*Incidence rate ratios (IRR) were determined to assess associations between antibody responses and overall incidence of clinical episodes for all individuals. Values represent unadjusted and adjusted ratios (age and location) ± 95% confidence intervals (CI). Significant associations are shown in bold.*

### ANTI-PfRipr ANTIBODIES ARE NOT ASSOCIATED WITH REDUCED RISK OF *P. falciparum* RE-INFECTION AND SYMPTOMATIC MALARIA

**FIGURE 4:**
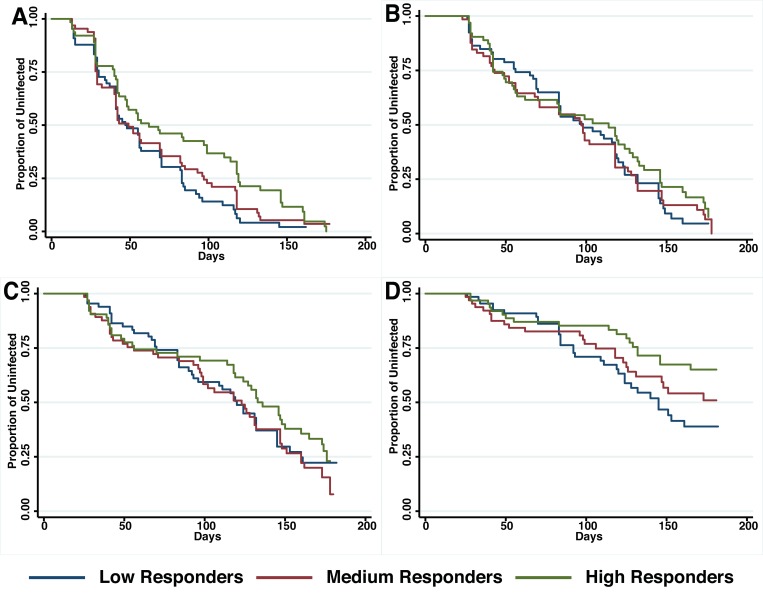
**Probability of remaining free of malaria re-infection amongst individuals with different anti-PfRipr antibody levels.** Time-to-first PCR-positive *P. falciparum* blood-stage infection **(A)**, LM-positive **(B)**, LM-positive with ≥500 parasites/μL **(C)** and LM-positive with ≥5000 parasites/μl **(D)** in individuals with low, medium, or high anti-PfRipr-specific antibody titers at study enrolment, as determined by Kaplan–Meier survival analysis. Log-rank was used to determine differences among responder groups, *p* = 0.01 between high and low responders with ≥5000 parasites/μl.

The relationship between anti-PfRipr antibody titers and time to reinfection of different parasite densities was investigated in Kaplan–Meier survival curves and a log-rank test was used to determine the difference between high, medium and low responders. **Figure 4** shows that only high responders (H vs L: *p* = 0.01) appeared to be protected from parasitemia of high density (>5000 parasites/μl) but not from moderate-density parasitemia (>500 parasites/μl) or re-infection. Before adjustment for confounders, high responders showed a reduced risk to develop a high-density infection (H vs L, HR: 0.49, 95% CI 0.28–0.86, *p* = 0.02). However, this association did not reach statistical significance after adjustment for age and location of the study participants (H vs L, HR: 0.38, 95% CI 0.33–1.05, *p* = 0.07).

The association of anti-PfRipr antibody levels with risk of experiencing a malaria clinical episode was also determined. The risk of developing clinical episodes (**Table 2**) as well as the overall incidence of malaria episodes throughout the 6 month follow-up period (**Table 3**) were similar amongst all responders, suggesting anti-PfRipr antibodies do not play an important role in clinical outcome.

## DISCUSSION

Antibodies that prevent *P. falciparum* invasion into erythrocytes are an important effector mechanism mediating immunity against blood-stage malaria parasites. Over the past 10 years, a large body of data has been generated towards understanding the molecular basis of merozoite invasion into the erythrocyte and many parasite antigens have been characterized. From those antigens, PfRh5 has been identified as an attractive vaccine candidate since it appears to be essential for parasite survival (Baum et al., 2009), has limited sequence polymorphisms and upon animal immunization induces broadly growth inhibitory antibodies (Douglas et al., 2011; Bustamante et al., 2013; Reddy et al., 2014). Consistent with those findings, immunization of rabbits with the PfRh5 recombinant protein described here induced strong antibody responses able to inhibit parasite growth across three different *P. falciparum* lines.

Previous studies suggested that PfRh5 is not highly immunogenic in humans exposed to malaria (Douglas et al., 2011) and that antibody responses to this antigen are rapidly lost at the end of high transmission season. However, a recent study conducted in Mali reported that naturally acquired anti-PfRh5 antibody responses predict protection from clinical malaria (Tran et al., 2014). Consistent with those findings, our study revealed strong associations between anti-PfRh5 antibody titers and protection from symptomatic malaria and high-density parasitemia but not re-infection. Moreover, anti-PfRh5 antibodies at study baseline were found to reduce the overall incidence of clinical episodes throughout the 6-month follow-up period.

PfRh5 exists in a membrane-associated complex with the cysteine-rich protein PfRipr. We have previously found antibodies to *E. coli* expressed C-terminal and N-terminal fragments of PfRipr inhibit merozoite attachment to the erythrocytes as well as *in vitro* parasite growth (Chen et al., 2011). Here immunization of rabbits with a baculovirus-expressed C-terminal fragment of PfRipr induced strong antibody responses, able to inhibit the *in vitro* growth of several *P. falciparum* lines to a similar level than that observed for anti-PfRh5 rabbit antibodies. Recombinant PfRipr was readily recognized by plasma from PNG participants in our cohort study. However, unlike anti-PfRh5 responses, antibodies against PfRipr showed only a modest association with protection from high-density parasitemia, which did not reach statistical significance after adjustment for confounder variables. Interestingly, despite the close association of PfRipr and PfRh5 on the surface of the merozoite, antibody responses against the two antigens appeared to be only weakly correlated (data not shown). To date, antibody responses to PfRipr have not been extensively investigated. Further work in other settings is required to determine whether this *Plasmodium* antigen is an important target of naturally acquired immunity to malaria.

There is an urgent need for a blood stage component in an anti-malarial vaccine to reduce parasite burdens responsible for morbidity and mortality in susceptible individuals. Development of a blood stage vaccine against malaria has been largely delayed mainly due to the high level of polymorphism in the few antigenic targets that were tested in clinical trials (Lyon et al., 2008; Ogutu et al., 2009). Thus it has been suggested that an effective blood stage vaccine should include more than one (preferably conserved) antigenic components to circumvent parasite phenotypic diversity. This report and others (Douglas et al., 2011; Bustamante et al., 2013; Reddy et al., 2014) demonstrated that immunization with PfRh5 results in the induction of strain-transcending parasite growth inhibitory antibodies. Similarly, we have recently found that immunization of rabbits with a highly conserved region of EBA-175 (region III–V) also induces potent, parasite strain-transcending growth inhibitory antibodies, which appear to be active at concentrations even lower than those described for anti-PfRh5 antibodies (Healer et al., 2013). Further studies are now required to investigate whether co-immunization with PfRh5 and the III–V EBA-175 region results in synergistic interactions and induces robust antibody responses required for the development of an effective malaria vaccine.

## AUTHOR CONTRIBUTIONS

Conceived and designed the experiments: Chris Y. H. Chiu, Jennifer K. Thompson, Julie Healer, Alan F. Cowman, and Diana Silvia Hansen. Performed the experiments and analyzed the data: Chris Y. H. Chiu, Jennifer K. Thompson, Julie Healer, and Diana Silvia Hansen. Contributed essential reagents/materials/analysis tools: Lin Chen, Connie S. N. Li Wai Suen, Peter M. Siba, Lin Chen, Ivo Mueller, and Alan F. Cowman. Interpreted data and wrote the paper: Chris Y. H. Chiu, and Diana Silvia Hansen.

## Conflict of Interest Statement

The authors declare that the research was conducted in the absence of any commercial or financial relationships that could be construed as a potential conflict of interest.
